# Gender differences in the use of an upper-extremity exoskeleton during physically and cognitively demanding tasks- a study protocol for a randomized experimental trial

**DOI:** 10.3389/fneur.2024.1401937

**Published:** 2024-10-18

**Authors:** Bettina Wollesen, Julia Gräf, Lasse Hansen, Anna Gurevich, Shirley A. Elprama, Andreas Argubi-Wollesen, Kevin De Pauw

**Affiliations:** ^1^Institute of Human Movement Science, Department of Human Movement and Exercise Science, Universitaet Hamburg, Hamburg, Germany; ^2^Institute of Movement Therapy and Movement-Oriented Prevention and Rehabilitation; German Sports University Cologne, Cologne, Germany; ^3^Institute of Biomechanics and Orthopaedics, German Sport University Cologne, Cologne, Germany; ^4^imec-SMIT, Vrije Universiteit Brussel & FARI-AI For the Common Good Institute, Brussels, Belgium; ^5^exoIQ GmbH, Hamburg, Germany; ^6^Human Physiology and Sports Physiotherapy Research Group (MFYS), Vrije Universiteit Brussel (VUB), Brussels, Belgium; ^7^Brussels Human Robotics Research Center (BruBotics), Brussels, Belgium

**Keywords:** muscle synergies, brain–body dynamics, wearable robotic systems, functional near infrared spectroscopy, overhead work

## Abstract

**Background:**

Upper limb exoskeletons are recommended to alleviate muscle fatigue, particularly in working conditions inducing musculoskeletal discomfort like overhead work. However, wearing an exoskeleton might introduce cognitive-motor interference, affecting performance. Understanding its neural impact and potential gender differences in design effects is crucial. Therefore, the aim of this study is to examine exoskeleton effects addressing cross-gender comparisons, and exploring the impact on cognitive and physical workload in real-world scenarios. The research questions address the impact of exoskeleton use on muscle synergies, upper body posture, cognitive resources, comfort/discomfort, acceptance and usability.

**Methods:**

The cross-sectional study integrates a multifactorial mixed-measure design. Participants are grouped by gender (female vs. male) and working condition (with vs. without exoskeleton). Motor performance and underlying neuronal correlates (fNIRS) will be analyzed. Based on an *a priori* sample size calculation, 80 participants (40 female/40 male) will be recruited. Working performance will be assessed by 1. Physical Performance Task (PILE task) and 2. Precision Task (following the Fitts paradigm), while body postures will be monitored with an Xsens motion capture system. Brain activation will be captured with an fNIRS system comprising 32 active optodes. Postural comfort/discomfort, acceptance, and usability will be reported via standardized questionnaires.

**Discussion:**

The study will gain insights into potential gender differences in exoskeleton use and will contribute to designing and optimizing the implementation of exoskeletons by considering muscle synergies, movement variability and cognitive resource allocation. Additionally, the study also highlights user discomfort, a crucial factor that could impede widespread adoption, particularly among females, in real-world scenarios.

## Introduction

The escalating demands within industrial settings, characterized by increased production volumes, repetitive tasks, and constrained postures, have led to a rising prevalence of musculoskeletal complaints, contributing to the emergence of musculoskeletal disorders (MSDs) (1). Musculoskeletal disorders, categorized by Van Eerd et al. (2) as inflammatory and degenerative conditions, pose significant challenges, particularly in the construction industry, where nearly 27% of employees faced such issues in 2017. The associated production downtime costs due to work absenteeism in Germany alone amounted to 17.2 billion euros (3).

Tasks performed at or above head height, common in industrial settings, pose substantial challenges and frequently lead to these MSDs. The use of exoskeletons, wearable external mechanical support structures designed to enhance user performance offer flexibility and performance enhancement, potentially reducing physical strains and preventing work-related diseases, especially MSDs (4). The application of exoskeletons involves human-machine interaction (HMI), encompassing both psychological and physical aspects. While previous studies have demonstrated positive effects of exoskeletons in terms of assistive support, there are challenges related to user muscular demands, altered posture, cognitive-motor interaction (CMI), and discomfort.

To gain more insights into these aspects, comprehensive approaches integrating the analysis of muscle activities and changes in muscle synergies in a combined measurement set-up including also kinematic data of body posture as well as brain dynamics to analyze CMI and the subjective impression of postural comfort or discomfort should be used. Following this idea, we summarized the state of the art of the main aspects of specific interacting parts of this approach in the next sections. These are: (1) Muscle activity and muscle synergies, (2) Movement/posture, (3) Cognitive-motor interference and (4) Postural Discomfort.

### Muscle activity and muscle synergies

In recent studies on (upper-body) exoskeleton use, researchers have already explored various aspects related to muscle activity, but with varying results (5–10). The need to support the back and shoulder muscles is particularly important for overhead and lifting activities (5–11). Studies have shown a reduction in muscular activation associated with exoskeleton use in the *M. deltoideus*, *M. Biceps brachii* during overhead tasks, and *M. Triceps brachii* (7, 8; 11). Different effects are observed in *M. Brachioradialis* and *M. pectoralis* major depending on the task (5, 8), and mixed results are presented regarding the activation of the *M. trapezius* but a consistent reduction in the M. Infraspinatus during exoskeleton-assisted overhead tasks (12, 13). Furthermore, the effectiveness of upper body exoskeletons in supporting back muscles presents varied outcomes for the *M. Latissimus dorsi* and *M. rectus* abdominis (9, 14, 15).

Regarding the interplay of the different muscle groups during exoskeleton use the muscle synergy extraction can be used to reduce degrees of freedom within high-dimensional datasets (16). After the initial proposal of the concept by Bernstein (17), several groups demonstrated that the central nervous system might simplify complex movements through the grouping of co-activated muscles into modular organizational units, called muscle synergies (18–20). A muscle synergy can be defined to be a vector specifying relative levels of muscle activation, with the absolute level of activation being modulated through a single neural command (21).

Among methodologies tested for muscle synergy extraction from surface electromyography data, non-negative matrix factorization (22) became generally accepted as the most suitable algorithm in locomotion tasks (23). Changes in muscle synergy number, synergy composition, or their respective activation profiles, has been observed within a wide range of subjects, e.g., due to cortical lesions (24), knee osteoarthritis (25, 26), or after total knee arthroplasty (27, 28).

Within the context of rehabilitation of stroke survivors, changes in muscle synergies have been also reported through the use of an active ankle-foot exoskeleton (29) and arm weight support (30). In an industrial setting with healthy participants, only one study investigated the effect of an active exoskeleton: The use of an active lumbar support exoskeleton during repetitive stoop lifting induced alterations within the synergies’ activation profiles, but only minimal alterations within the relative levels of muscle activation (31). No studies have yet investigated the effect of an industrial upper limb exoskeleton on muscle synergy composition, yet understanding these effects can play a vital role in understanding the complex interplay between user and device.

In addition, limited evidence exists regarding gender differences in muscle activity during exoskeleton use, with some studies indicating higher muscular demands and discomfort for females during overhead and carrying tasks (6, 32, 33). Moreover, to our knowledge, no additional research has specifically examined gender disparities in the use of upper body exoskeletons for tasks involving overhead movements or carrying/lifting activities.

### Movement/posture

Besides muscle activation, the upper-body exoskeletons should be able to optimize movements and reduce physical stress. A full-body analysis during an overhead task was executed by Latella et al. (34) showing a significant reduction in internal full-body moments ranging from 66 to 86%. Furthermore, internal joints loads were found to be decreased as well in the shoulder and trunk area. However, this decrease was compensated by general increase in the load on the legs because internal strains intuitively transfer to the lower body, particularly the hips.

### Cognitive-motor interference

Next, to the movement aspects, the use of exoskeletons involves a complex interplay between users and the support system, encompassing psychological and physical elements in human-machine interaction (HMI) or cognitive-motor interference. This interaction is often observed in scenarios requiring the concurrent execution of tasks, involving cognitive and/or motor resources and leading to cognitive-motor interference [CMI; (35)]. CMI, characterized by performance decline in dual-task situations, may incur dual-task costs (36). Various models, rooted in the principles that performance quality is tied to processing activity quantity, and this processing is inherently limited, provide insights into the concept of CMI (37). Initial studies have delved into cognitive load associated with exoskeleton use across various contexts, unveiling CMI effects that impact pace and reaction times [e.g., (38)]. Evidence indicates that exoskeleton use requires heightened effort in terms of motor adaptation and neurocognitive control. Factors such as cognitive load, cognitive-motor process complexity, demographic variables, and exoskeleton characteristics contribute to CMI. Recent reviews corroborate the impact of upper-limb exoskeletons on cognitive workload and physical performance. In a study by Taygi et al. (33), neurophysiological adaptations during exoskeleton use were investigated. Their research uncovered variations in neural activity and functional connectivity within the primary motor cortex, offering valuable insights into the influence of exoskeletons on the nervous system. In the realm of exoskeleton research, a meticulous exploration of the discomfort aspect has yielded valuable insights from several studies. For example, Otten et al. (9) presented noteworthy findings, revealing a tangible decrease in overall task effort with the implementation of the Lucy exoskeleton. This reduction suggests a potential ergonomic advantage associated with exoskeleton use.

### Postural discomfort

Despite all potential benefits of exoskeleton use, De Bock et al.'s (39) investigation added complexity to the understanding of discomfort, uncovering shifts and heightened frustration among exoskeleton users. Interestingly, this discomfort was accompanied by a simultaneous reduction in temporal workload, indicating a nuanced interplay of factors influencing user experience. In a separate study, Borrel Rubio and Mora Quiles (40) delved into the ergonomics of a shoulder exoskeleton during overhead assembly tasks with findings of a reduction in shoulder joint moments, indicating positive strides in enhancing user comfort. The integration of the NASA TLX questionnaire further enriched the analysis, revealing diminished perceived physical demand and a marginal reduction in mental effort. Contrary, Weston et al. (41) found slightly higher overall discomfort while using an upper-body exoskeleton during overhead tasks. Additionally, Giustetto et al. (42) found that the effect of the exoskeleton on comfort/discomfort differs in different parts of the body. For example, the exoskeleton was able to reduce discomfort in the lumbar spine region while increasing discomfort in the chest and feet. These studies therefore show that it may not be possible to increase the comfort level for all body regions.

Depending on these interdisciplinary considerations, an exoskeleton might influence cognitive and motor performance via different pathways: it would be possible that cognitive resources are given free due to physical assistance with decreased physical demands and reduced exhaustion or reflecting motor control theories, the additional use and wearing of an exoskeleton needs to be integrated into automatized movements and therefore might need more cognitive resources.

Therefore, the aim of this study is to advance our understanding of exoskeleton effects by conducting inclusive research, addressing cross-gender comparisons, and exploring the impact on cognitive and physical workload within real-world scenarios. Based on these objectives, we define the following questions with the focus on gender differences:

1. Are there gender effects of using an upper body exoskeleton for both tasks regarding:
  - Muscle synergies?
  - Upper body posture?
  - Cognitive resources?
  - Comfort/discomfort, acceptance and usability?
2. Are there gender effects of using an upper body exoskeleton for both tasks under previous physical fatigue for:
  - Muscle synergy?
  - Upper body posture?
  - Cognitive resources?

**Figure 1:**
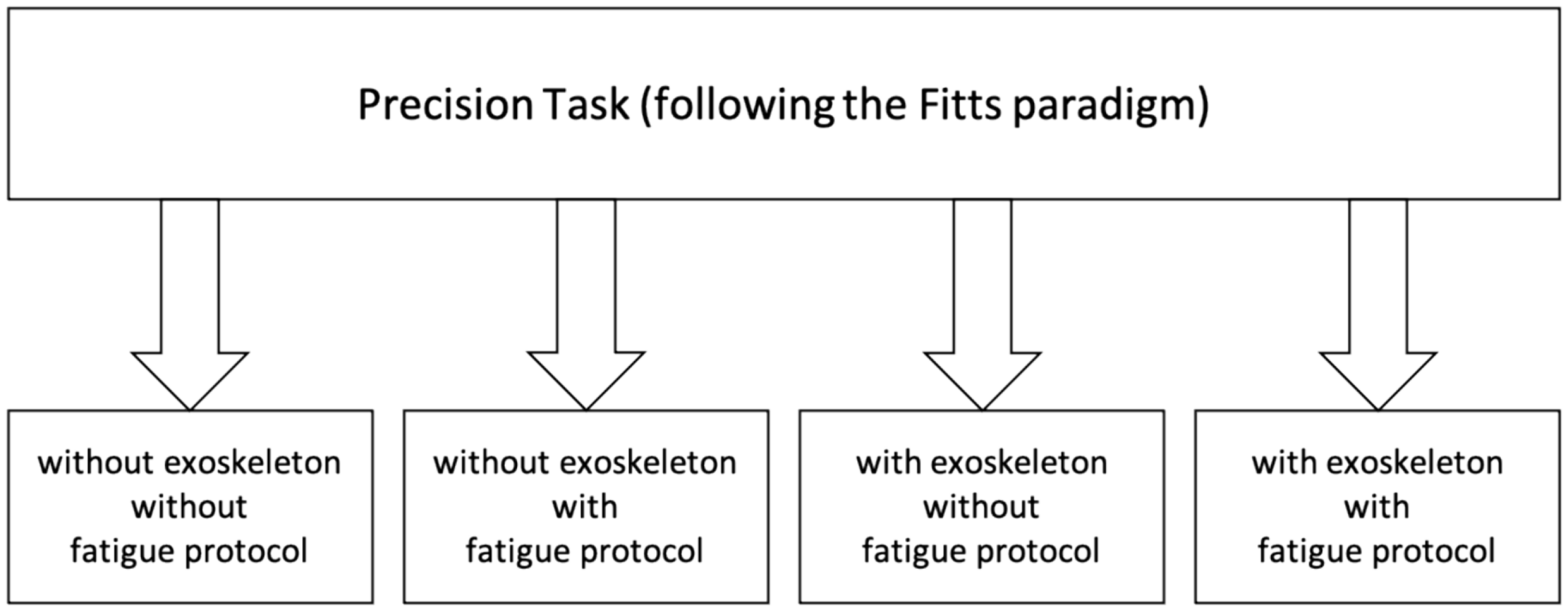
Study flow of the precision task (nailing following the Fitts paradigm).

**Figure 2:**
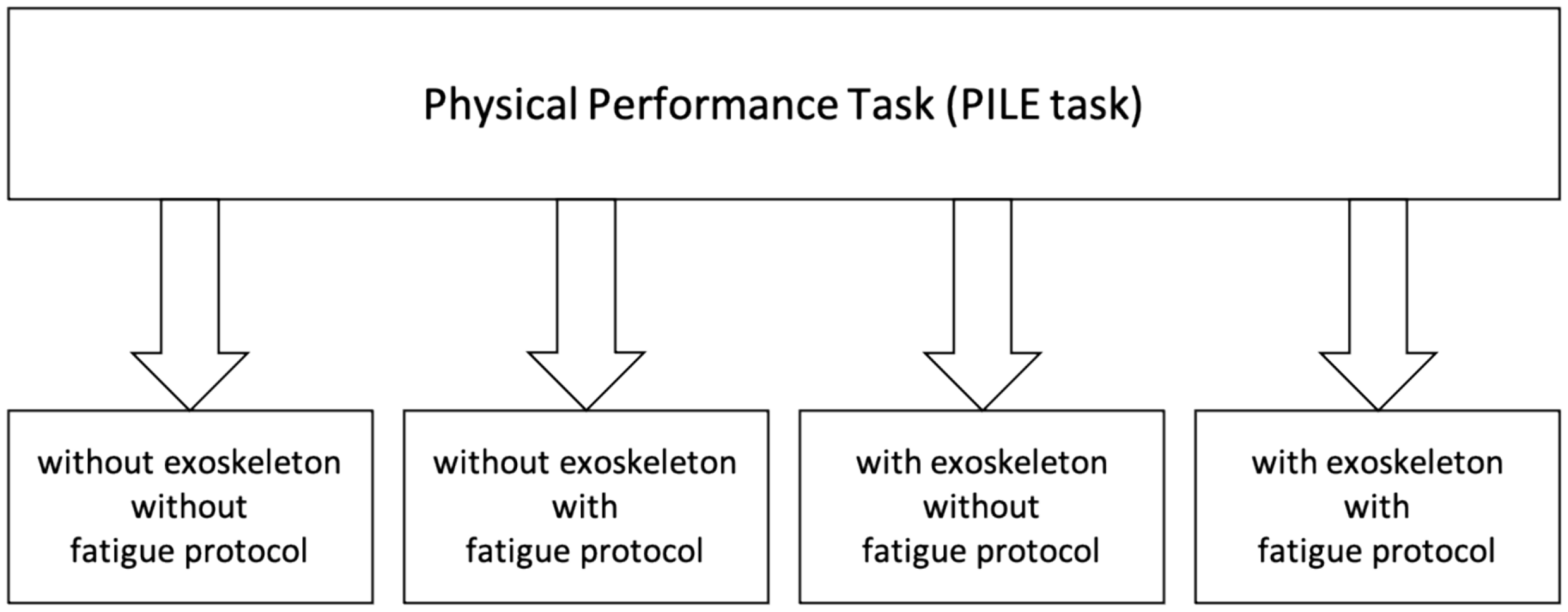
Study flow of the physical performance task (PILE task).

The research questions will be answered using a randomized crossover study design (*cf.* Figures 1, 2). Participants of each gender will be randomly assigned to different sequences of interventions, ensuring a balanced distribution of order effects. This design allows each participant to serve as their control, contributing to internal validity.

## Methods and analysis

### Trial design

This study includes a randomized experimental cross-sectional design comparing males and females (parallel groups). This means that each participant runs through each scenario in a randomized order.

### Study setting

The study will be conducted as a monocentric study at the University of Hamburg (Germany). For the experiments a specific workstation is installed to secure the ecological validity.

### Exoskeleton being used

This study will conduct the experiments using the battery powered, pneumatic exoIQ (name of the company) S700 active Exoskeleton. The S700 is purposefully designed to support during manual tasks in and above shoulder height and is commercially available from 2023. It can be adjusted to fit both men and women from between 1.65 m up to 2.05 m body height and with various body types. Both, the amount of supporting torque and the characteristics of its application during use (torque curve) can be altered to the demands of user and task. These alterations are being stored on the device via presets. For each task, a specified preset will be used and the amount of support torque will be adjusted to the individual need.

### Eligibility criteria

Participants should be within the age range of 18–45 years, between 1.65 m and 2.05 m body height and do not have any chronic physical or mental health disorders.

The experiments will be executed by trained sports scientists with at least a Bachelor degree.

### Experimental set-up/intervention

The experimental design includes two physical tasks (1. Physical Performance Task and 2. Precision Task) in four randomized conditions (with and without exoskeleton and with and without prior physical fatigue; *cf.* Figures 1, 2):

1. Physical Performance Task (PILE task) at shoulder height:Participants will perform the Physical Performance Task at shoulder height, both with and without the exoskeleton. This task aimed to assess the impact of the exoskeleton on physical performance under normal working conditions.

1. Precision Task (nailing following the Fitts paradigm) at overhead height:The Precision Task involves overhead nailing activities following the Fitts paradigm (43, 44) (three predefined templates into which the nails must be precisely inserted.), simulating tasks requiring precision and skill. Participants will execute this task with and without the exoskeleton, allowing the evaluation of the exoskeleton’s influence on tasks demanding fine motor skills.

Randomization will be done by the online program www.randomizer.org.

### Experimental procedure

#### Precision task (nailing following the Fitts paradigm)

The Precision Task consists of simulating overhead nailing work during a work process. The nails are shot overhead into a device using a nail gun. The process can be compared, for example, with screwing or drilling processes in industrial or craft areas. The Einhell TE_CN 18-Solo cordless nailer will be used.

The Precision task includes the following sequence [*cf.* (43)]: The subjects stand under the setup. To determine the height of the fixture, the test subjects will be asked to bring the pistol into contact with the wooden plate with the elbow at acromion level. The time will be measured by the start signal of the measuring supervisor and the test person begins the task. The aim is to try to hit the target (Fitts task) as quickly and accurately as possible with the nail gun so that a visible imprint will be made on the paper. If a field is missed, the measuring supervisor instructs the participants to retrieve it. As soon as a template is completed, the time will be stopped, errors noted and it is replaced by the next template (Fitts task). The standardized break between the three patterns is 30 s. After another start signal, the task and timing will be repeated until all three templates are completed. There are two conditions, which are performed at random with the exoskeleton and without the exoskeleton.

#### Physical performance task (PILE task)

The PILE task, which was developed by Mayer et al. (45), is a functional assessment tool for measuring manual lifting ability. It involves lifting a weighted box in four movement cycles from a starting position to two predetermined heights: from the floor to hip height and from the hip to shoulder height. The task assesses cardiovascular endurance, overall lifting capacity, psychophysical fatigue of the trunk and limbs and motivational factors through repeated lifting cycles. During the task, the weight is increased after each successful cycle, starting at 4 kg for women and 6 kg for men. The total test duration is approximately 10–15 min. The aim is to assess lifting ability for specific heights, taking into account the maximum weight lifted, time to abort and heart rate at abort. The aim of the task is to calculate the work performed and the strength. The PILE task shows a high test–retest reliability with correlation coefficients of r = 0.87 for lifting at hip height and r = 0.93 for lifting at shoulder height (*p* < 0.001). Termination criteria include psychophysiological factors, aerobic capacity exceeding 85% of maximum heart rate, and safety concerns related to exceeding a predetermined weight limit. Normative values for test performance are calculated based on age, height and weight, with age influencing the calculation of aerobic capacity and height determining the tolerable lifting capacity. For this study purpose, we only use the hip to shoulder height task.

#### Physical fatigue session

A fatigue session takes place, following a randomized order, e.g., before the PILE task or before the nailing task. The fatigue session will be performed in 3 sets, with a 2-min calf raise and a 2-min front raise with weight per set, with the front raise in the second set only being performed for the second minute. The weight of the men is 5 kg and that of the women 2.5 kg. There will be a break of 60 s between the sets. During the exercise, a metronome runs at 60 bpm, and the front and calf raises are to be performed to this beat. Before the start of the fatigue session, the maximum body height will be determined individually on the anthropometer by the test person standing upright and bringing their feet into plantar flexion. This determined height should be reached during each exercise. The level of fatigue will be determined using EMG power spectral shifts (Fast Fourier Transform) in combination with a visual analog scale of peripheral fatigue.

Questionnaires on acceptance and discomfort of the conditions with exoskeleton use will be filled out after the experimental procedure.

### Outcomes

To achieve these aims, the following tests and measurement instruments will be included in the experimental set-up:

#### Motion analysis

3-D motion analysis will be used to answer the research questions 1 and 2 regarding the effects of upper body posture with and without the use of the exoskeleton during both tasks (*cf.* research question 1 b) as well as comparing both tasks under previous physical fatigue (2 b).

Motion analysis based on video recordings, and 3-D motion analysis via Xsens will be carried out.

- Video recordings will take place to analyze participants’ body posture through a specified movement observation sheet and evaluation based on a scoring system [OWAS; (46)].
- 3-D motion analysis will be performed via Xsens MVN 2018, a three-dimensional kinematic motion measurement system. The angular measures of the body (shoulder girdle, arms, wrists, upper and lower back, legs) are evaluated. Body movements are recorded via 17 inertial sensors that enable a three-dimensional analysis of the body segments in three dimensions (47).

#### Muscle activity

Muscle activity will be measured to address research questions 1and 2, which investigate the effects of using an upper body exoskeleton on muscle synergies during task performance, both in general (1b) and under conditions of prior physical fatigue (2b).

In the realm of biomechanical research, the analysis of muscle activity through surface electromyography (sEMG) is a pivotal methodology, particularly when scrutinizing the upper extremity muscles exposed to loading during overhead activities. This comprehensive investigation extends to an array of key muscles, especially in the context of upper extremity activities and overall body stability. The included muscles encompass *M. Biceps brachii*, *M. Triceps brachii caput longum*, *M. Brachioradialis* as prime movers of the forearm, as well as *M. deltoideus*, *M. pectoralis* major, *M. trapezius descendens*, *M. Latissimus dorsi* and *M. Infraspinatus* as movers around the shoulder. Ultimately the *M. rectus* abdominis will be included due to its role in postural stability. For the accurate measurement of muscle activity, adherence to the established guidelines is decisive. The “SENIAM Guidelines,” as articulated by Hermens et al. (48), outline the standardized protocols for non-invasive assessment. By following these guidelines, the research endeavors to ensure precision and reliability in the collection and interpretation of sEMG data, thereby advancing our understanding of the intricate interplay of muscles during dynamic upper extremity movements.

#### Muscle synergies

The methodology behind calculating muscle synergies is focused on analyzing sEMG data of muscles involved in multijoint movements. The aim is to build groups of synergistic muscles with distinct temporal activation patterns and individual muscle weightings. The method described to be most favorable to identify these groups is the non-negative matrix factorization of time-series data (23). The approach involves a comprehensive examination of muscle activity and their interactions during various physical activities.

#### Brain hemodynamics using fNIRS

Functional near-infrared spectroscopy (fNIRS) will be used to answer research questions 1 and 2, which investigate the effects of using an upper-body exsoskeleton on cognitive resources during task performance, both in general (1c) and under conditions of prior physical fatigue (2c).

Functional near-infrared spectroscopy (fNIRS) is a non-invasive, optical imaging technique for measuring brain activity in the cerebral cortex based on the interactions between neuronal activity, cellular metabolic processes, and increased blood flow (neurovascular coupling).

During cognitive, perceptual, and motor processes, there is increased neuronal activity in certain regions of the brain. As a result, there is an increased demand for energy and oxygen, which is met by the influx of oxygen-enriched blood. This leads to a relative increase in the concentration of oxygenated hemoglobin (O2Hb) compared to deoxygenated hemoglobin (HHb). To measure this change, infrared-emitting emitter diodes (transmitters) and light-sensitive detectors (receivers) are attached to the skull with a cap. The fNIRS measurement is based on the projection of infrared light (700–900 nm) from a source diode into the tissue under the scalp and the measurement of the intensity of the backscattered light with detectors. Due to the different optical properties, the concentration of O2Hb and HHb can be determined from the amount of backscattered light. By evaluating changes in oxygen consumption over time, conclusions can be drawn about the activity of cortical areas during the processing of certain tasks, emotional reactions and cognitive information.

#### Speed accuracy trade offs (Fitts task)

The Fitts task is a standard motor control experiment in which participants move quickly between two targets of different sizes and distances (44). The aim is to assess the speed and accuracy of the target movements. The difficulty of the task is determined by a difficulty index, which is calculated based on the size and distance of the targets. The researchers manipulate the task conditions to investigate factors that influence movement time and accuracy. Key measures include movement time and error rate, which provide insight into the trade-off between speed and accuracy in motor control. The Fitts task is often used in human-computer interaction and to assess motor skills.

The Borg rating of perceived exertion is used together with the SUS and the NASA-TLX questionnaires to answer research question 1d, investigating the effects of using an upper body exoskeleton on comfort/discomfort, acceptance and ease of use during task performance.

#### Subjective perceived exertion (Borg-scale)

The subjective assessment of the perceived exertion is carried out by using the 15-point Borg scale (6–20) after each test session. 6 points equal very very low exertion, and 20 points equal very very high exertion (49). The assessment refers to the overall perceived exertion and specific body regions, such as the neck, shoulders, arms, upper and lower back, and legs. A chart is shown for this purpose.

#### Subjective workload assessment questionnaire

The Subjective workload assessment questionnaire (NASA-Task Load Index, NASA-TLX) addresses the technical part of the experiment, capturing workload at multiple levels, regarding the interaction with the exoskeleton: mental demands, physical demands, time demands, performance, effort, and frustration (50).

#### System usability scale (SUS)

The System Usability Scale (SUS) provides a reliable tool for measuring usability. Comprising 10 questions with response options ranging from “Strongly Agree” to “Strongly Disagree.” This tool facilitates the assessment of diverse products and services, encompassing hardware, software, mobile devices, websites, and applications.

#### User acceptance

We have added a few questions on user acceptance. The topics of these questions are from Elprama et al. (51) and they are based on the framework of exoskeleton acceptance by Elprama et al. (52) with 5-point scale answers, mostly ranging from “Strongly Agree” to “Strongly Disagree.”

### Participant timeline

The study will be conducted over two separate days to mitigate potential carryover effects and ensure the avoidance of additional fatigue symptoms. On each day, participants will undergo the assigned conditions for both tasks in a randomized order, minimizing the impact of confounding variables. The sequence of conditions will be counterbalanced across participants to control for order effects.

First, the participants are welcomed and informed in writing and verbally about the aim and conduct of the study. This is followed by the recording of the anthropometric data and personal data. Then the preparations for the surface electromyography begin, including the application of the electrodes and the pre-tests (MVC). The suit for the Xsens sensors is then applied and a calibration is carried out. Finally, the sensor and the calibration for the fNIRS are carried out.

The first session then begins, depending on which task was selected first for the person. The person is then either fitted with the exoskeleton and begins the first task (Physical Performance Task or Precision Task) or performs this without the exoskeleton. If the fatigue protocol is already planned for the person at the beginning based on the randomization, this is carried out without the exoskeleton before the start of the task.

After completion of the first task, an individual break is taken before the second task begins. After completing both tasks, the person is asked to complete the questionnaires.

Due to the activity of overhead work, which already involves increased exertion because of the non-ergonomic posture, and the additional activity of screwing, physical fatigue may increase. Therefore, physical exertion is subjectively documented via the Borg scale (49) after each test session. Additionally, test sessions are spread over 2 days.

**Figure 3:**
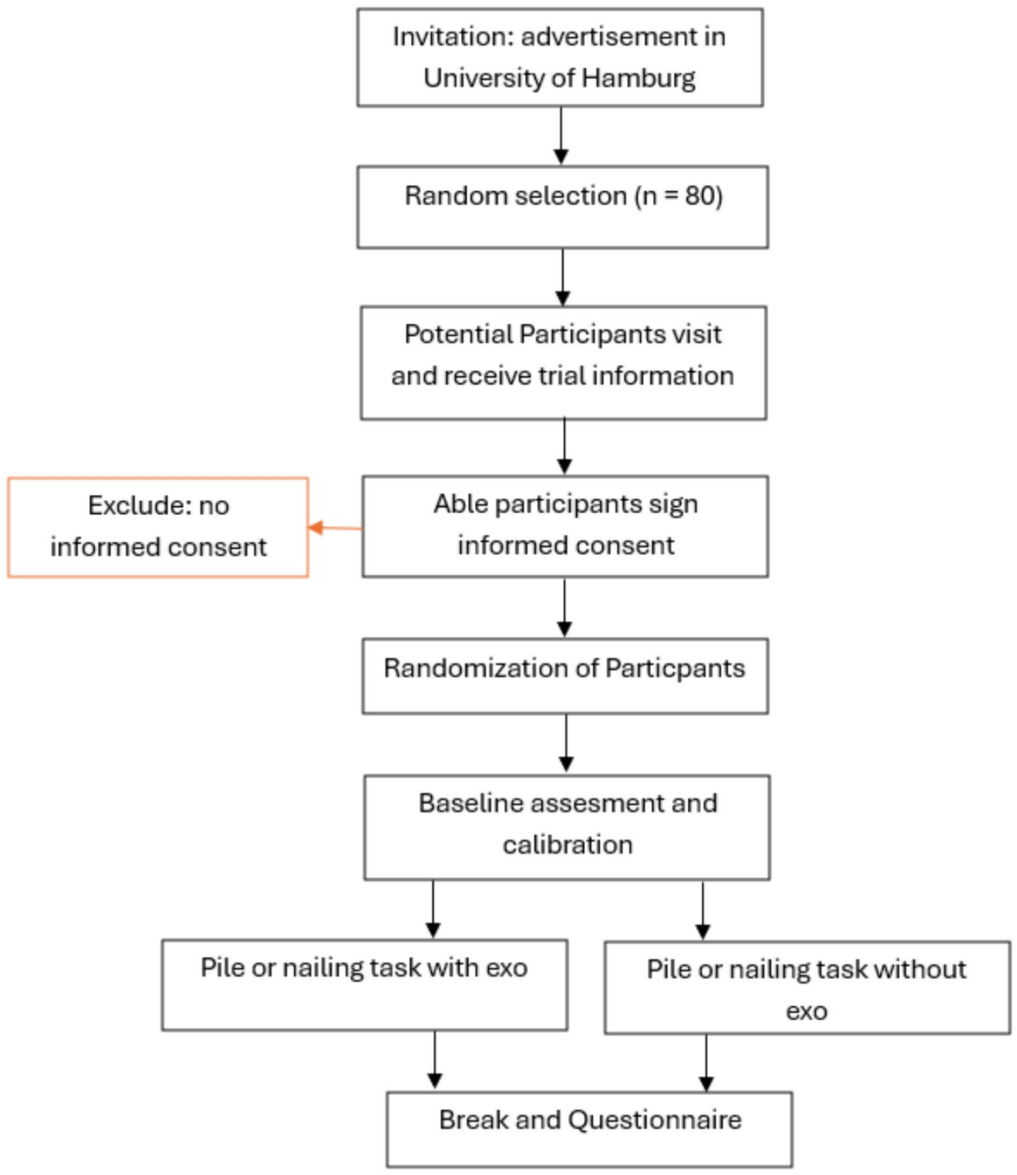
Participants time schedule.

Individual breaks will be provided between each test session due to possible mental and physical exertion during the tests (Figure 3).

### Sample size

The sample size was calculated on the outcome of muscle activity using G*power (*a priori*, ANOVA with repeated measures and interaction effect) with 2 groups (male/female), 2 tasks (Physical Performance Task, Precision Task), 2 conditions (with/without exoskeleton), and 2 fatigue protocols (with/without fatigue protocol) estimating an effect size of 0.25, an alpha error rate of 0.05 and a statistical power of 0.80, resulting in *N* = 36 participants for each group. With an estimated drop of 20% we will include a total of *N* = 80 (40 female/40 male) healthy participants with male and female participants equally.

### Recruitment

Recruitment will take place through notices at the University of Hamburg as well via advertisements in local newspapers. By the use of a snowball concept, we will ask all participants to advertise the study and provide additional participants. In previous experiments, we have found that this strategy was particularly useful to find more participants.

### Data collection

The data collection will take place in the premises of the University of Hamburg, Department of Human Movement Science. Before the study is carried out, the participants are instructed not to do any strenuous sport, as this can already mean pre-fatigue and thus falsify the data. The participants are also informed in advance of the study that physical and mental fatigue may occur during and after the study. In addition, care is taken to ensure that the study is carried out at similar times of day so that a drop in performance over the course of the day can be taken into account.

### Data management

Our data collection tools include movement analysis via Xsens, muscular activation via sEMG, cognitive strain via fNIRS, subjective demand via Borg Scale and comfort of using the exoskeleton via questionnaire. The following personal information of the participants is required in the study: Gender, age, height and body mass, handedness, use of visual aids, and anthropometric measurements for calibration of the 3D motion analysis (body length, hip height, foot length, etc.).

All objective and subjective measurement instruments are synchronized and stored anonymously for evaluation. The data analysis is thus blinded for the study management, except for the analysis of the video recordings. Neither the participants nor the study management are blinded during implementation.

The study coordinator JG and the study supervisor BW will have data access.

#### Data protection

The data collected within the context of the study following the declaration of consent of the participants are covered by confidentiality and the provisions of data protection law. The study prioritizes data confidentiality and security through initial pseudonymization by generating a code followed by anonymization, in accordance with the University of Hamburg’s data protection guidelines. Participants in the study can be identified by pseudonyms linked to their personal details, allowing them to request access to their personal scores via the secure coding list. Only the research team will have access to this list, and participants can request the removal of their personal data until it is deleted. Personal data together with the coding list and research data will be stored separately, with access restricted to the principal investigator and authorized staff, and no third-party transfers will be permitted. The coding list will be maintained until data evaluation is completed.

#### Harms

Wearing an exoskeleton can cause a feeling of discomfort (53), however it is not expected that an exoskeleton will harm a participant.

In the event that the participant shows or expresses strong symptoms of exhaustion or discomfort, the study is terminated without any disadvantage to the participant. If necessary, the study is repeated on another day.

#### Informedness

The participants are fully informed about the background and the objectives of the research project (respective subject information).

Additionally, to enhance participant retention, we will keep them engaged and informed by sharing the study results.

#### Consent

Participation in the studies is voluntary. The participants are verbally and in writing informed and have sufficient time to understand the content of the study. They can withdraw from the study at any time without giving reasons and without incurring any disadvantages.

In the event of withdrawal of consent during the project period, the data already collected will be deleted upon request. In this case, the pseudonymization will be canceled by the respondent himself/herself, and the disclosure of the code, as otherwise the data set cannot be determined.

The subjects will sign the attached consent forms before the start of the study. These contain detailed participant information, information about OEMG studies as well as information about video recordings and fNIRS studies.

### Data monitoring

The whole author group will be involved into the data monitoring hosting meetings once a month. Following the data collection, the raw data will be reviewed, and possible elimination of poor data will be made (cleaning of the data set). Standardized procedures will be applied to the sEMG data sets, the fNIRS data set, and the questionnaires.

The raw data of the 3D motion analysis will be extracted, processed, and evaluated via MATLAB (Matlab R2019b, Massachusetts, USA).

The video recordings are reviewed to ensure that all measuring instruments are synchronized.

### Statistical analysis

All data will be processed via SPSS 29.0 and evaluated using suitable statistical procedures at the significance level *α* = 0.05. These statistical procedures depend on concrete questions and include variance analyses as well as descriptive procedures.

For the analysis of the movement data (Xsens), repeated measures ANOVA will be carried out with regard to upper body flexion, lateral flexion and rotation with a comparison between the different scenarios. Furthermore, we will conduct Bonferroni *post-hoc* tests for between-subject analysis regarding differences between (1) using an exoskeleton, and (2) fatigue protocol. In addition, an SPM analysis will provide information on the sections of the activity in which a change over time can be detected.

For the analysis of muscular activation, an ANOVA with repeated measures is also used for the comparisons between the scenarios, as well as Bonferroni *post-hoc* tests for between-subject comparison. In addition, a Statistical Parameter Mapping (SPM) analysis can also be carried out here, which illustrates the deviations over time.

The subject-specific muscle synergies will be classified through k-means clustering using the Hartigan-Wong algorithm independently for each group and condition. For statistical analysis of muscle weights between groups two-way analysis of variance (ANOVAs) will be carried out, which in case of statistical significance will be followed by post-hoc comparisons through pairwise t-tests with Benjamini-Hochberg adjusted *p*-values. For all muscle weightings that show significant differences, effect sizes (Cohen’s d) will be calculated. Furthermore, the activation patterns will be analyzed through the calculation of center of activity, as well as the full width at half maximum (FWHM). Additional discretes for analysis can be number of synergies per subject and percentage of unclassifiable synergies.

All objective data recordings will be synchronized via LSL and processed with specific MATLAB pipelines.

For the analysis of the cognitive data (fNIRS), as well as the subjective perceived exertion via the Borg scale, an ANOVA with repeated measures is carried out in each case.

## Discussion

The aim of this study is to increase the understanding of the effects of exoskeleton use, especially with regard to cross-gender comparisons while exploring cognitive and physical workload within real-world scenarios. The main research question will address the gender differences effects of using an upper body exoskeleton in the dual task working situations regarding muscle synergies, upper body posture, cognitive resources, comfort, discomfort and usability. Moreover, an additional focus will lay on the effects of a physical fatigue protocol on the proposed outcomes.

The randomized crossover study design (*cf.* Figures 1, 2) ensures a balanced distribution of order effects and the high number of participants that will be included in the study is unique according to the combined and comprehensive methods (muscle synergy concept and fNIRS).

### Gender differences

With respect to the research questions, we hypothesize that potential gender differences will be observed within the muscle synergies. We think that the exoskeleton use will reduce shoulder muscle activity significantly [e.g., (54)] but first studies suggest that women might have less muscle activation in the shoulder muscles while wearing an exoskeleton (33). On the other hand, with respect to the design and the weight of the exoskeleton, women potentially could use different movement patterns to manage the tasks (56) due to differences in biomechanical and anthropometric trunk muscle geometry of females and males (56, 57). We anticipate that women show more bending backwards movements including a higher compensatory muscle activation in the abdominal and neck muscles. However, this idea has to be proven within our experimental design.

Moreover, as women are merely not integrated into the iterative designing processes of exoskeletons, yet, we propose more discomfort and less usability in the ratings by the women. This discomfort especially will be reported for the shoulder region (41).

In line with the cognitive resource theories (36), this discomfort might lead to more attentional resources spending on the discomfort and less resources available for the working DT situation. This will increase the cognitive-motor interference especially in the DT situation and could be observed with more cortical activation in the prefrontal cortex [*cf.* (35)]. Moreover, the working accuracy will decrease (58).

### Fatigue effects

Regarding physical fatigue we suppose that firstly the overall gender effects might be reduced. One potential explanation lies in the idea that the additional required motor resources after fatigue will reduce the discomfort effect. According to fatigue it has to be assumed that the task management requires more muscle activity that can be reduced by the exoskeleton use (59). As this is relevant for both genders, the muscle activity and accompanying synergies might converge. Nevertheless, it is unclear if the weight of the exoskeleton affects muscle activity between men and women differently.

However, the organizing of body movement after fatigue needs more executive control in the motor cortex (60) and this will lead to less attentional resources available for the detailing recognition of the discomfort. This hypothesis will be proven by the analysis of the fNIRS data, regarding a potential shift from parietal to frontal cortex activation. This mechanism should be equal for male and female participants and might only be affected by age (61).

Moreover, as more resources for postural control and motor adjustments will be required after fatigue, the cognitive-motor interference in the DT condition will rise while movement accuracy will decrease. If the exoskeleton use will reduce this effect, it needs to be examined within our experimental design. On the other hand, the physical fatigue could also increase the proposed differences in upper body postures (more bending backwards of the women). This hypothesis would lead to increased muscle activity in the supporting muscles and might change muscle synergy patterns.

In summary, the knowledge of this study will contribute to refining and optimally implementing exoskeletons, with a focus on reducing fatigue and mitigating musculoskeletal risks, while considering the nuances of movement variability and cognitive resource allocation.

Furthermore, we would like to emphasize that the study also highlights the challenges, such as user discomfort and frustration, which can hinder the widespread adoption of this technology in real-world scenarios, especially for females.

The innovative focus lies on task and gender-specific variability including the muscle synergy approach. Analyzing muscle synergies can provide a nuanced understanding of muscle coordination and function, as well as the effects of interventions such as fatigue protocols or wearing an exoskeleton on these interactions.

Regarding the nature of this study protocol, we are aware that the potential outcomes we hypothesize are only speculative and need to be confirmed with the data we will collect. Moreover, with respect to the study design, there might be aspects of muscle fatigue and the interplay with cognitive resources that cannot be answered with this study design (e.g., what happens if different forms of additional cognitive tasks will be added or if we also integrate a mental fatigue protocol). Nevertheless, the described complex study design of this protocol will gain a lot of relevant insights to provide guidelines for optimizing the design of these robotic applications.

### Possible pitfalls and limitations

Most pitfalls in exoskeleton research occur due to limitations of the device. Either, the exoskeleton is a research prototype, which makes it oftentimes prone to failure. Or in commercially available (and therefore more robust) exoskeletons, their applicability for certain tasks are limited, placing constraints on support torque modifications for a heterogenous pool of test subjects or remain difficult to fit to certain test subjects (often times women due to differences in body shape in comparison to men).

In order to avoid these pitfalls, we intend to use the commercially available exoskeleton exoIQ S700. It is specifically designed to provide support in the task being assessed (overhead work) with numerous torque support adjustments. Moreover, its comparatively wide range of size adjustments (shoulder width, back length, arm length) and textiles, that are designed to fit both genders, should suffice to significantly mitigate these possible issues.
